# Sustainable Removal of Diclofenac from Aqueous Effluents Using PET Waste-Derived Activated Carbon: Experimental and Simulation-Based Process Optimization

**DOI:** 10.1021/acsomega.5c09493

**Published:** 2026-01-21

**Authors:** Amanda R. de S. Araujo, Daniela R. da Costa, Diego Diniz, Gabriela U. Manzoni, Gustavo V. Olivieri, Andreia de A. Morandim-Giannetti

**Affiliations:** Department of Chemical Engineering, 42511 Centro Universitário FEI, Av. Humberto de Alencar Castelo Branco, 3972 São Bernardo do Campo, São Paulo, Brazil

## Abstract

Activated carbon was obtained from polyethylene terephthalate (PET). Three activating agents were tested (KOH, ZnCl_2_, and H_3_PO_4_) while evaluating effluents containing sodium diclofenac, and an investigation into the optimal conditions for effluent treatment was conducted. H_3_PO_4_ showed a higher adsorption capacity (34.06 mg·g^–1^) to sodium diclofenac when used in a 2:1 ratio (mass of H_3_PO_4_: mass of carbon). MEV analyses show that the materials exhibit mesopores and macropores on the surface of the activated carbon obtained (15.3–120 nm). BET analyses revealed that H_3_PO_4_ activation produced the largest surface area (542.97 m^2^·g^–1^), corroborating the morphological observations and explaining the superior performance in sodium diclofenac adsorption. Four kinetic and two isotherm adsorption models were also tested to fit the experimental data. The kinetic and isotherm adsorption tests indicated that this capacity can be maximized when the process is carried out at 25 °C for 25 min (estimated adsorption capacity of 200 mg·g^–1^). The kinetic and isotherm adsorption models enabled simulations to replicate the batch process and to prospect the industrial application of an adsorption column.

## Graphical abstract

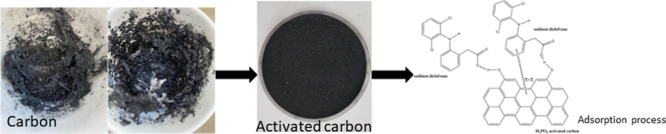

## 1 Introduction

The growing global consumption of plastic packaging, estimated at approximately 16.5 million tons per year, mainly composed of polyethylene terephthalate (PET), has led to a significant increase in improperly discarded waste in terrestrial and aquatic environments.^1–5^ To decrease this environmental impact, extensive research has focused on recycling PET into high-value products such as fuels, new polymers, resins, plasticizers, concrete additives, and fibers, through depolymerization processes including hydrolysis, methanolysis, ammonolysis, aminolysis, glycolysis, pyrolysis, and carbonization.^6–9^ Mapping this research landscape is crucial for identifying scientific gaps and guiding the sustainable valorization of PET residues.

In this context, the bibliometric analysis using the Scopus database (keywords combined through Boolean operators) revealed more than 73,000 studies published in the past five years on activated carbon synthesis. However, only 135 of these specifically address activated carbon derived from PET waste, highlighting a significant research gap, particularly in studies focused on wastewater treatment. This imbalance highlights the potential of converting PET waste into activated carbon as a sustainable and underexplored approach for producing environmentally relevant functional materials.

Activated carbon is widely used in various environmental processes, particularly in the treatment of industrial effluents generated by the chemical and pharmaceutical industries.^10–13^ The pharmaceutical industry, in particular, produces large volumes of wastewater containing persistent and bioaccumulative compounds that contribute to aquatic toxicity.^14^ Among these pollutants, sodium diclofenac, a common analgesic and anti-inflammatory medication, has been frequently detected in surface waters due to its extensive use and incomplete removal during conventional wastewater treatment.^15–17^ Developing cost-effective and sustainable technologies capable of efficiently removing such pharmaceutical contaminants is, therefore, an urgent environmental challenge.

According to Nieto et al. (2017), conventional effluent treatments remove between 22 and 39% of the sodium diclofenac in the effluents.^18^ This treatment involves a combination of coagulation, biodegradation, photodegradation, flocculation, sedimentation, filtration, and adsorption processes. Therefore, adsorption is an alternative for treating effluents from the pharmaceutical industry, hospitals, and health-related areas, as traditional treatment processes are inefficient due to the unique properties of these compounds.^16–20^ Within this framework, adsorption based on activated carbon has emerged as one of the most promising post-treatment technologies for wastewater remediation.

Several materials can be used in adsorption, e.g., activated carbon, chitosan, and lignocellulosic materials.^21–23^ Activated carbon has been widely studied, as it can be obtained from various sources, including wood, biomass, coffee beans, coconut shells, vegetable waste, and polymers such as PET.^13,24^ However, it is essential to emphasize that a literature search using the Scopus platform yielded only 38 articles on the treatment of effluents containing diclofenac with activated carbon, demonstrating that few studies have been conducted using this technique, which offers high efficiency and low cost. When considering the use of PET for the production of activated carbon, this number is even more restricted, with only six published articles, which reinforces the importance of conducting studies in this area aimed at the recycling of polymeric materials, especially for the treatment of effluents containing pharmaceutical products, an area in which there are no evident studies involving the recycling of PET, the production of activated carbon, and its application in effluent treatment.

This trend aligns with the broader context of circular economy strategies, focused on waste valorization through pyrolysis and chemical activation processes. In this sense, activated carbons synthesized from various raw materials, including nutshells, agricultural byproducts, and polymeric waste, have shown high specific surface areas and relatively high adsorption capacities. Furthermore, adsorption-based processes have been identified as one of the most effective post-treatment methods for removing pharmaceutical micropollutants from wastewater, demonstrating efficiencies above 90% when using granular or powdered activated carbon. These studies, together, demonstrate the growing scientific and technological relevance of activated carbon derived from PET as a sustainable and efficient material for environmental remediation.^25–29^

In this context, this work investigated the process for producing activated carbon from PET packaging waste and its use for the adsorption of sodium diclofenac from synthetic effluents. Furthermore, a process simulation based on kinetic and isotherm models was performed using the Aspen Plus software to prospect a continuous adsorption system. This work differs from previous studies by integrating experimental data with process modeling to support industrial scalability.

## 2 Experimental Section

### 2.1 Materials

Analytical-grade reagents were used to prepare all solutions and adsorbents. Potassium hydroxide (≥98%), hydrochloric acid (36.5%–38%), and phosphoric acid (98%) were acquired from Dinamica Reagentes (Brazil). Zinc chloride (97%) was purchased from Cinética (Brazil). Sodium diclofenac was obtained from Merck (USA).

### 2.2 Activated Carbon from PET Packaging

The material (PET package) was cut into 0.5 cm × 0.5 cm pieces and carbonized in porcelain crucibles with lids in a muffle furnace. An initial temperature of 50 °C was used, reaching a plateau of 500 °C in 45 min (10 °C·min^–1^). After this period, the material was maintained at this temperature for 2 h, and then the temperature was increased to 600 °C using a temperature ramp of 10 °C·min^–1^. After reaching 600 °C, the material remained at this temperature for 1 h, then cooled naturally. Afterward, the carbon was submitted to the activation process.

Three activating agents (potassium hydroxide, zinc chloride, and phosphoric acid) were evaluated to determine which provided the best results for the further adsorption of sodium diclofenac in synthetic effluents. The activating agents (activation with ZnCl_2_, H_3_PO_4_, or KOH) were added separately at a mass ratio of 1:2 (activating agent to carbon). The mixtures were stirred for 2 h, filtered, and then dried at 100 °C for 24 h to remove moisture. Subsequently, they were subjected to a calcination step at 500 °C for 1 h and washed with an HCl solution (2.5%) or water to remove residues of the activating agent. The activated carbon was subjected to a grinding step in a ball mill for approximately 4 h, washed with distilled water to clear its pores, and characterized via X-ray diffraction (XRD), BET surface area analysis, particle size analysis by laser diffraction, energy dispersive spectroscopy (EDS), and scanning electron microscopy (SEM).

### 2.3 Characterizations

Particle size distributions were analyzed using a Microtrac Bluewave laser particle size analyzer (Montgomeryville, USA), which measures particle sizes from 0.01 to 2800 μm. The microparticles were analyzed in dry mode in triplicate. The X-ray diffraction analyses were performed in a Shimadzu XRD-7000 X-ray diffractometer (Japan). The operating voltage and current were maintained at 40 kV and 35 mA, respectively, and the angular range was 3°–135°. The diffraction spectra were collected using the θ–2θ method. The samples were scanned in the 2θ range of 5°–35° with a step size of 0.015°. The crystallinity index (CI) was determined using the ratio of the crystalline area to the total area (eq 1).^30^

1 $$CI=\frac{crystalline\;area}{total\;area}\times 100\%$$

The SEM-EDS analyses were performed using a JEOL JSM-7500F (Japan) high-efficiency scanning electron microscope, using carbon tape as the substrate. The images were obtained using an accelerating voltage of 2.00 kV, and an increase of 5000 times was used. The materials’ BET surface area and porous properties were measured using a Micromeritics ASAP 2020c Plus (Accelerated Surface Area and Porosimetry System) (USA) at 77 K, according to the mathematical treatment proposed by Brunauer, Emmett, and Teller (BET method) from nitrogen gas adsorption experiments.

### 2.4 Bach Adsorption Experiment

During the adsorption process, the best activator was evaluated. 0.05 g of each activated carbon obtained (activation with ZnCl_2_, H_3_PO_4_, or KOH) was added to 25 mL of a sodium diclofenac solution (75 ppm). The mixture was stirred in an orbital incubator shaker (Inova 43, Eppendorf International, Germany) at 250 rpm for 30 min. All tests were performed in duplicate. The resulting solution was filtered and evaluated by ultraviolet spectroscopy in a Kasuaki UV/vis spectrophotometer using a wavelength of 274 nm.^31^ The adsorption capacity was determined using eq 2.

2 $$q=\frac{\left(c_{i}-c_{f}\right)\times V}{w}$$

Where: *q* represents the adsorption capacity, *c*_i_ the initial concentration, *c*_f_ the final concentration, *V* the sample volume, and *w* the mass of the adsorbent.

The ideal dosage of the adsorbent (activated carbon) was determined. The experiments were duplicated using 0.01, 0.02, 0.03, 0.04, and 0.05 g of the adsorbent. All the systems were submitted using the same procedure described previously. The adsorption kinetics and the influence of temperature on the adsorption process were studied.

During the kinetic study, 0.03 g of activated carbon was added to a 25 mL solution of sodium diclofenac (75 ppm). The systems were stirred in an orbital incubator shaker at 250 rpm at 25 °C. The adsorption times were 0.17, 0.5, 1, 2, 5, 10, 15, 20, 25, 30, 40, 50, and 60 min. The samples were filtered and characterized by UV/vis spectroscopy using the same procedure described previously. The pseudo-first-order, pseudo-second-order, Weber and Morris intraparticle diffusion, and Elovich models were used to perform the kinetic studies (Table 1). All tests were performed in duplicate.

**Table 1:** Evaluated Kinetic Models and Their Parameters

model	equation	parameters
pseudo-first order	ln(*q*_e_ – *q*_*t*_) = ln *q*_e_ – *k*_1_ × *t*	*q*_e_ = equilibrium adsorption capacity (mg·g^–1^)
		*k*_1_ = kinetic constant (min^–1^)
		*q*_*t*_ = adsorption capacity (mg·g^–1^) at time *t* (min)
pseudo-second order	$\frac{t}{q_{t}}=\frac{1}{k_{2}{\times q}_{e}^{2}}+\frac{t}{q_{e}}$	*q*_e_ = equilibrium adsorption capacity (mg·g^–1^)
		*q_t_* = adsorption capacity (mg·g^–1^) at time *t* (min)
		*k*_2_ = second-order kinetic constant (g·min^–1^·mg^–1^)
Weber and Morris intraparticle diffusion	*q*_*t*_ = *k*_int_ × *t* ^0.5^ + *C*	*q_t_* = adsorption capacity (mg·g^–1^) at time *t* (min)
		*k*_int_ = intraparticle constant (mg·g^–1^·min^–0.5^)
		*C* (the linear coefficient) = resistance to diffusion (mg·g^–1^)
Elovich model	$q_{t}=\frac{1}{\beta }\times \ln\left(\alpha \times \beta \right)+\frac{1}{\beta }\times \ln\left(t\right)$	*q_t_* = adsorption capacity (mg·g^–1^) at time *t* (min)
		α = initial adsorption rate (mg·g^–1^·min^–1^)
		β = desorption constant (g·mg^–1^)

During the study of adsorption isotherms, 0.03 g of activated carbon was added to 25 mL of sodium diclofenac solutions with different concentrations (75, 100, 150, 200, 250, 300, 400, and 500 ppm). The adsorption process was carried out at various temperatures (20, 25, 30, 35, and 40 °C) for 25 min, with a rotation speed of 250 rpm. The samples were filtered and characterized via UV/vis spectroscopy in the region of 274 nm. Langmuir and the Freundlich isotherms (Table 2) were used to study the diclofenac adsorption process and its distribution on the surface of the activated carbon and in the solution during adsorption equilibrium. All tests were performed in duplicate.

**Table 2:** Adsorption Isotherms Used and Their Parameters

model	equation	parameters
Langmuir	$\frac{C_{e}}{q_{e}}=\frac{1}{q_{0}\times K_{L}}+\frac{C_{e}}{q_{o}}$	*K*_L_ = equilibrium constant between the adsorption and desorption process (L·mg^–1^)
		*q*_0_ = maximum adsorption capacity (mg·g^–1^)
		*C*_e_ = adsorbate concentration (mg·L^–1^)
		*q*_e_ = adsorption capacity (mg·g^–1^)
Freundlich	${\log q}_{e}={\log K}_{F}+\frac{1}{n}\times {\log C}_{e}$	*C*_e_ = adsorbate concentration (mg·L^–1^)
		*q*_e_ = adsorption capacity (mg·g^–1^)
		*K*_F_ = adsorption capacity of Freundlich (mg^(1–1/^ *^n^* ^)^·L^1/^ *^n^* ·g^–1^) *n* = constant, which tends to lie between 1 and 5 for favorable adsorptions

Thermodynamic studies were also conducted to determine the thermodynamic parameters Δ*H*° (enthalpy), Δ*S*° (entropy), and Δ*G*° (Gibbs free energy), providing insight into the spontaneity and heat change during the adsorption process of sodium diclofenac on activated carbon. The parameters were calculated by applying the experimental values obtained from the adsorption experiments of sodium diclofenac using the van’t Hoff eqs 3 and 4

3 $${\Delta G}_{ads}=-R\times T\times \ln K_{C}$$

4 $$\ln K_{C}=-\frac{{\Delta G}_{ads}}{R\times T}=\frac{{\Delta S}_{ads}}{R}-\frac{{\Delta H}_{ads}}{R\times T}$$

where: Δ*G*_ads_ represents the variation of the energy of Gibbs (kJ mol^–1^), Δ*H*_ads_ the enthalpy variation (kJ mol^–1^), Δ*S*_ads_ the entropy variation (kJ mol^–1^ K^–1^), *K*_C_ the equilibrium constant, *R* the universal gas constant (0.008314 kJ mol^–1^ K^–1^), and *T* the process temperature (K).

### 2.5 Process Simulation

The adsorption process was simulated in the software Aspen Plus (V14) by treating the adsorption phenomenon as analogous to a chemical reaction, i.e., sodium diclofenac (in solution) to sodium diclofenac (adsorbed). The kinetic model inserted into the software was based on the pseudo-second-order kinetic model and the Langmuir isotherm adsorption model, which were previously fitted to the experimental data. The Electrolyte Non-Random Two Liquid (ELECNRTL) was employed as the thermodynamic model for the simulations.

The RBatch equipment was used to simulate the transient configuration, aiming to replicate the experimental conditions tested in the kinetic study and to validate the adaptation of the customized kinetic expression based on the software requirements to describe it. The tested conditions were a temperature of 25 °C, a volume of aqueous solution of 25 mL, an initial concentration of sodium diclofenac in the solution of 75 ppm, a mass of adsorbent (activated carbon with H_3_PO_4_) of 0.03 g, and a time of 60 min. The profile of the adsorption capacity over time was generated and compared with the experimental data.

After the transient validation, a preliminary simulation of a continuous adsorption column was evaluated based on an analogy to a tubular reactor (RPlug equipment) filled with the adsorbent (acting as the “catalyst” of the reactor). The temperature was 25 °C, and sodium diclofenac’s feed concentration in the aqueous solution was 75 ppm. A sensitivity analysis was performed to evaluate the percentage of adsorption capacity (%*q*), defined as the percentage of adsorbed sodium diclofenac divided by the mass of adsorbent, using eq 5

5 $$\%q=\frac{100}{w}\times \left(\frac{\dot{w_{i}}-{ẇ}_{f}}{\dot{w_{i}}}\right)$$

where: *w* represents the mass of adsorbent, ${ẇ}_{i}$ the mass flow rate of sodium diclofenac in the solution feed stream, and ${ẇ}_{f}$ the mass flow rate of sodium diclofenac in the solution exit stream.

The varied conditions in the sensitivity analysis were the mass of the catalyst (ranging from 50 to 1000 kg) and the mean residence time (varying from 0.25 to 1.67 h, equivalent to volumetric flow rates ranging from 1000 to 150 m^3^·h^–1^).

## 3 Results and Discussion

### 3.1 Obtaining the Activated Carbon

The carbonization step was initially performed, and the material was submitted for characterization via X-ray diffraction. The results show the complete absence of crystalline residues and impurities after the heat treatment of the samples. This characteristic is noted by the low presence of signals in random band locations. Additionally, X-ray diffraction revealed a single band located approximately at 2θ = 23°. This band represents the (002) graphitic carbon reflections. They are typical of carbonized materials, indicating the presence of disordered carbon crystalline structures in the obtained material and polymer residue (Figure 1).^32,33^ These results are consistent with existing patterns identified in the works by Bratek et al. (2013) and Kaur et al. (2019).^34,35^

**Figure 1:**
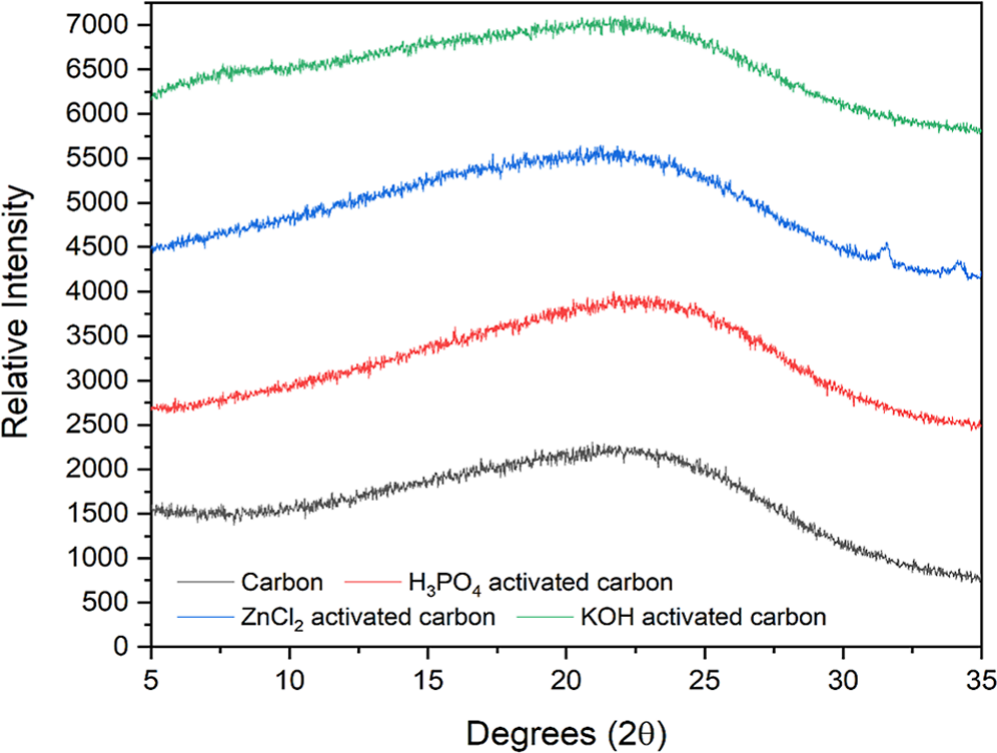
Diffractograms of carbon, H_3_PO_4_-activated carbon, KOH-activated carbon, ZnCl_2_-activated carbon, and standard activated carbon.

The activation using potassium hydroxide, zinc chloride, and phosphoric acid was evaluated. The adsorbents were characterized via particle size determination, X-ray diffraction, scanning electron microscopy, energy dispersive spectroscopy, and adsorption tests to verify the efficiency of the activator. The particle size results indicate that the activated carbon treated with KOH has an average particle size of 65.33 μm. In comparison, the one that started with ZnCl_2_ had an average particle size of 37.64 μm, and the carbon activated with H_3_PO_4_ showed an average particle size of 35.29 μm. Compared to the commercial activated carbon (64.67 μm), the one that showed the closest size was the one activated with KOH.

The XRD patterns (Figure 1) of all activated carbons exhibit broad diffraction features, characteristic of predominantly amorphous carbon structures. The broad band observed in the 5–20° 2θ region and the low-intensity peak around ∼23–25° 2θ indicate a low degree of structural ordering. In the case of H_3_PO_4_-activated carbon, a slightly broader diffraction profile and reduced peak intensity were observed, suggesting a higher degree of structural disorder and amorphization induced by phosphoric acid activation. According to Shamsudin et al. (2016), high concentrations of H_3_PO_4_ in the activation process increase concavity.^36^ This study demonstrates that using lower temperatures for activation yields better morphological structures. The diffractogram of the sample of KOH-activated carbon presented a similarity with that of the non-activated carbon. This behavior is also supported by the study conducted by Farma (2019), which showed a curve with similar behavior^32^ when using ZnCl_2_. It was possible to observe in its XRD pattern signals at 2θ between 30° and 36°. The study by Hidayu and Muda (2016) shows that these signals are characteristic of the zinc particles in the carbon pores.^37,38^

It was also possible to obtain the percentage of crystallinity of the samples of activated carbons (H_3_PO_4_-activated carbon = 8.31%; ZnCl_2_-activated carbon = 10.30%; KOH-activated carbon = 13.30%, commercial activated carbon = 11.54%, and carbon = 11.14%). The crystallinity of the carbon is made of heteroatoms bound to the carbon during the activation process, or in the case of non-activated carbon. This crystalline peak indicates the presence of oxygen bound to its surface during the carbonization process.

SEM analyses show that the non-activated carbon has a smooth and homogeneous surface. After its activation, pore formation can be observed, and the EDS analysis confirms the identification of elements present in the activated materials (Figure 2). The KOH had a more significant impact on the structure of the coal, compared to the other activators, presenting a surface of larger and heterogeneous pores due to activation. The ZnCl_2_ presented a rough surface, whereas the activation with H_3_PO_4_ resulted in a smooth and homogeneous surface with the formation of porous concavities. A highly porous structure was also observed for the materials activated with KOH and H_3_PO_4_; however, activation with ZnCl_2_ altered the surface, resulting in a rougher and less porous aspect. This characteristic is essential for explaining the low adsorption of carbon with this agent. The activated carbon treated with H_3_PO_4_ exhibits a structure with smaller and more defined pores, as compared to the other activators. This characteristic is essential since adsorption is enhanced on surfaces with a better pore structure.

**Figure 2:**
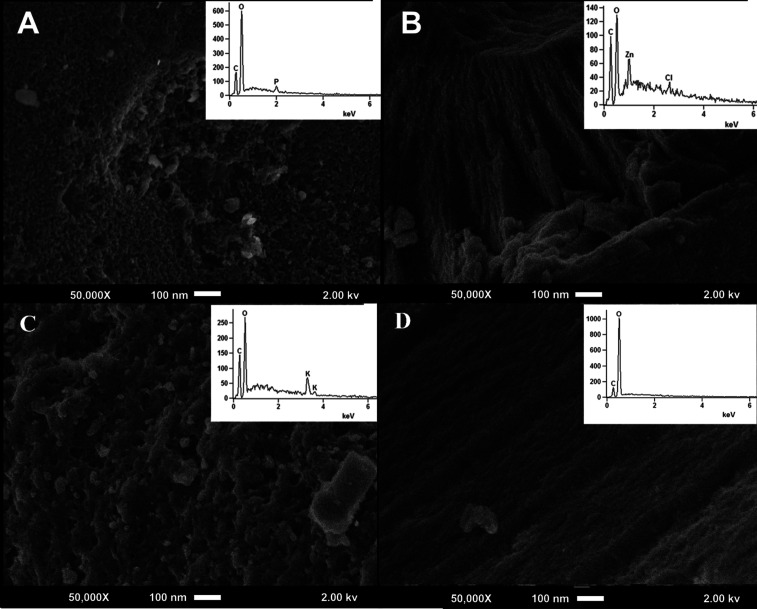
SEM images and EDS spectra for (A) H_3_PO_4_-activated carbon, (B) ZnCl_2_-activated carbon, (C) KOH-activated carbon, and (D) non-activated carbon.

BET surface area analysis provided quantitative confirmation of the morphological differences observed by SEM. The specific surface areas obtained were 226.82, 429.93, and 542.97 m^2^·g^–1^ for ZnCl_2_, KOH, and H_3_PO_4_ activated carbons, respectively. The significantly larger surface area of the sample activated with H_3_PO_4_ indicates that phosphoric acid is the most effective activating agent for generating an extensive network of micro- and mesopores (15.3–120 nm). Furthermore, the larger surface area correlates directly with the greater adsorption capacity observed in the removal of sodium diclofenac.

The higher adsorption performance of the H_3_PO_4_-activated carbon is therefore attributed to its textural properties, as confirmed by BET surface area measurements and SEM observations, rather than structural features inferred from XRD. The significantly larger specific surface area and the well-developed micro- and mesoporous network, as evidenced by nitrogen adsorption–desorption analysis, provide a suitable basis for correlating activation conditions with porosity and adsorption efficiency.

In addition, adsorption tests of sodium diclofenac were conducted to evaluate the activating agent that provided the most effective activated carbon for treating effluents containing this compound (Figure 3). Analysis of the results showed that phosphoric acid yielded the most effective reduction in diclofenac concentration. This factor can be explained by the fact that diclofenac exhibits better adsorption on both positively and negatively charged surfaces; repulsive interactions can occur due to the presence of the COO^–^ group in the diclofenac molecule (Figure 4). Activation of the carbon by H_3_PO_4_ presents a surface enriched by phenolic groups (C_Aromatic_–OH), which are sources for hydrogen bonds. The study by Mokhati et al. (2021) demonstrates that the primary mechanisms of diclofenac adsorption on H_3_PO_4_-activated carbon occur through physicochemical interactions and electrostatic forces between the molecules, with minimal impact on the morphological structure of the carbon (as observed in the SEM test) and other ionic bonds.^39^

**Figure 3:**
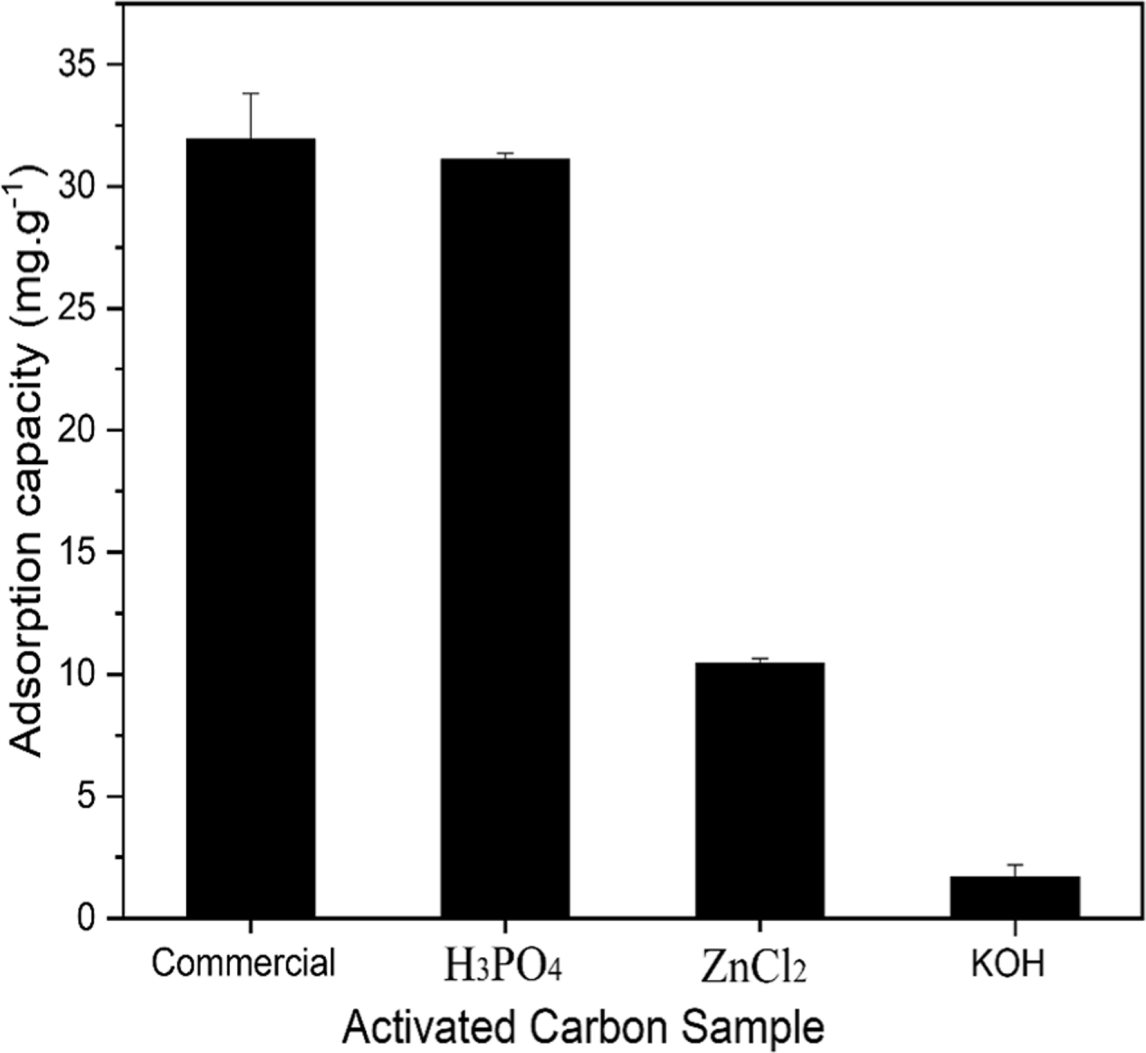
Adsorption capacity of activated carbon obtained with different activating agents and commercial activated carbon.

**Figure 4:**
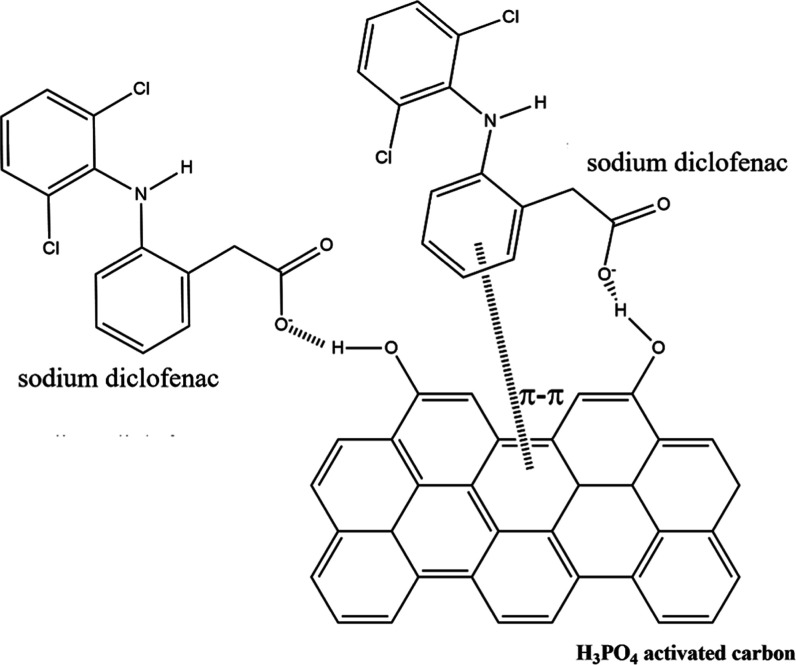
Adsorption mechanism of sodium diclofenac on activated carbon with H_3_PO_4_.

Beyond the chemical interactions promoted by surface functionalization, the porosity generated during H_3_PO_4_ activation plays a decisive role in the adsorption of diclofenac molecules. The SEM results, corroborated by the BET results, indicate that phosphoric acid induces dehydration and cross-linking reactions that promote the development of porosity in the structure, markedly increasing the accessible surface area and enhancing molecular diffusion within the carbon matrix. These pores facilitate the coexistence of physical and chemisorptive processes: micropores contribute to high adsorption capacity through van der Waals and π–π interactions, whereas mesopores enhance intraparticle diffusion and mass transfer, thereby preventing steric hindrance for larger organic molecules, such as diclofenac. Furthermore, the combined presence of oxygenated and phosphate functional groups within the porous framework enhances hydrogen bonding and electrostatic attraction, resulting in cooperative adsorption phenomena that strengthen overall removal efficiency.^40–43^

### 3.2 Adsorption Process

After determining the best activator (H_3_PO_4_), a comparison was made between different proportions of phosphoric acid based on the mass of carbon. It can be observed that the best result was obtained using a ratio of 2:1 (Figure 5A). The maximum dosage was 0.03 g of activated carbon (Figure 5B).

**Figure 5:**
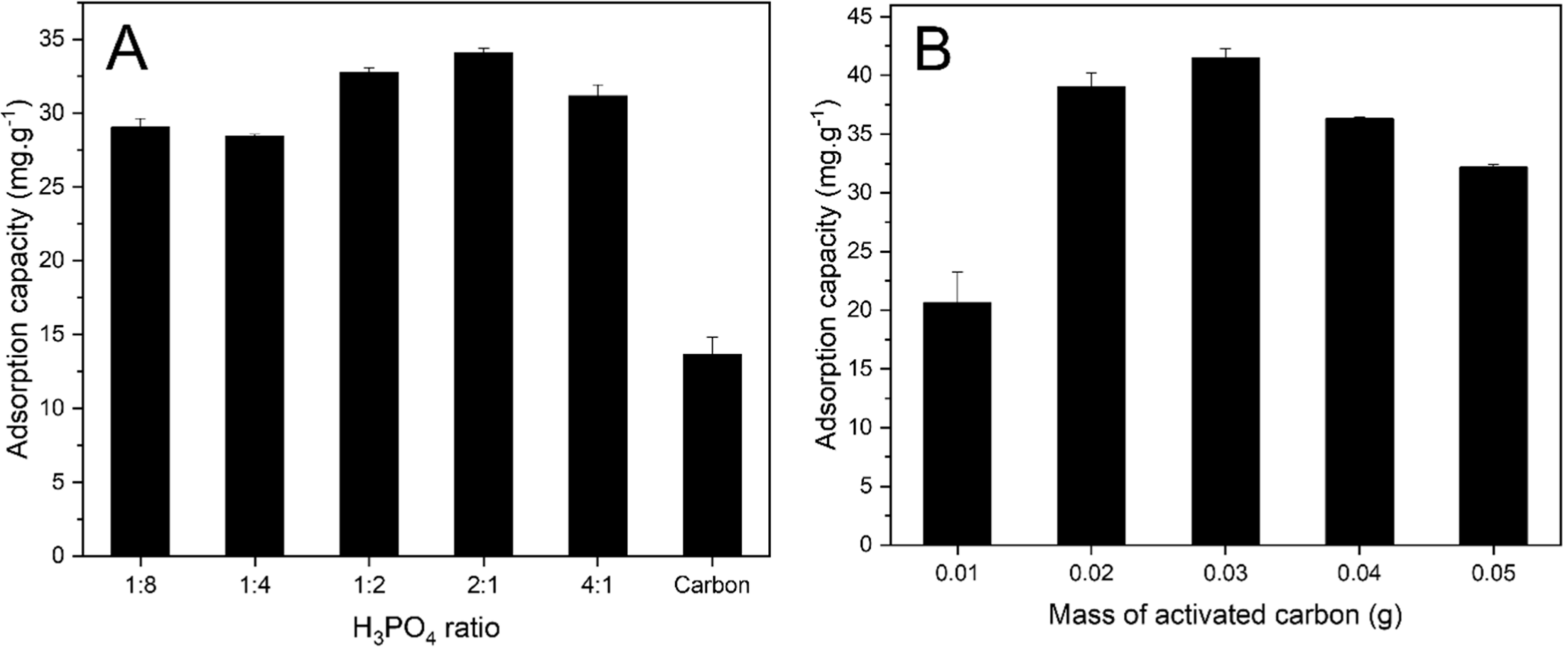
(A) Adsorption capacity as a function of the H_3_PO_4_ ratio used in the activation and (B) adsorption capacity as a function of the activated carbon mass.

Kinetic studies were subsequently performed to determine the optimal treatment time for the effluent. All data were evaluated using the pseudo-first-order, pseudo-second-order, Weber and Morris intraparticle diffusion, and Elovich models (Table 3). The pseudo-second-order model yields the best results in the adsorption kinetics of diclofenac with PET-activated carbon, indicating that the kinetic constant of the reaction is approximately 0.01 g·mg^–1^·min^–1^. The adsorption capacity at equilibrium is 41.49 mg·g^–1^.

**Table 3:** Kinetic Parameters for H_3_PO_4_-Activated Carbon

model/parameter	H_3_PO_4_-activated carbon
pseudo-first order	
*q*_e_ (exp) (mg·g^–1^)	39.80
*q*_e_ (calc) (mg·g^–1^)	24.03
*k*_1_ (min^–1^)	0.05
*R* ^2^	0.98
pseudo-second order	
*q*_e_ (exp) (mg·g^–1^)	39.80
*q*_e_ (calc) (mg·g^–1^)	41.49
*k*_2_ (g·min^–1^·mg^–1^)	0.01
*R* ^2^	1.00
Weber–Morris	
*q*_e_ exp (mg·g^–1^)	39.80
*q*_e_ calc (mg·g^–1^)	39.77
*C* (mg·g^–1^)	10.18
*K*_d_ (mg·g^–1^·min^–0.5^)	6.99
*R* ^2^	0.96
Elovich	
*q*_e_ exp (mg·g^–1^)	39.80
*q*_e_ calc (mg·g^–1^)	40.10
α (mg·g·min^–1^)	137.54
β (g·mg^–1^)	0.17
*R* ^2^	0.97

Other works that utilized activated carbon for the adsorption of the drug in question also demonstrated pseudo-second-order kinetics, as observed in studies by El Naga et al. (2019) and Daouda et al. (2021).^44,45^ Studies conducted by Avcu et al. (2021) and Kumar et al. (2022) also found that the pseudo-second-order model best described the adsorption process.^16,21^

In addition, this was verified by evaluating the Weber and Morris model data, which showed that the curve does not intersect its origin, indicating that the adsorption studied is not limited by intraparticle diffusion. For the Elovich model, it is possible to confirm that the process that occurs consists of chemisorption.^46^ Studies of the isotherms (Table 4) indicate that the adsorption process is favorable, as the *R*_L_ values (separation factor) between 0 and 1 were obtained. This fact suggests that the activated carbon tends to adsorb diclofenac until it reaches a specific equilibrium capacity, after which, even with an increase in the concentration of the solute in the solution, there will be no increase in the amount adsorbed on the surface of the adsorbent.

**Table 4:** Langmuir and the Freundlich Isotherm Parameters for H_3_PO_4_-Activated Carbon

model	temperature (°C)	20	25	30	35	40
Langmuir	*q*_max_ (mg·g^–1^)	212.77	200.00	172.41	192.31	196.08
	*K*_L_ (L·g^–1^)	0.01	0.01	0.02	0.02	0.01
	*R*_L_^Max^	0.81	0.81	0.72	0.66	0.77
	*R*_L_^Mín^	0.22	0.22	0.12	0.11	0.21
	*R* ^2^	0.99	0.98	0.99	0.93	0.99
Freundlich	*n*	2.04	2.60	2.66	2.11	2.00
	*K*_F_ (mg^(1–1/^ *^n^* ^)^·L^1/^ *^n^* ·g^–1^)	9.87	20.04	16.93	9.51	8.83
	*R* ^2^	0.98	0.82	0.98	0.94	0.94

It was possible to verify that the adsorption process occurs in monolayers with a uniform free energy change and no interaction between the adsorbed species. In this case, the adsorbent was not able to adsorb to the surface of the adsorbent, but it was able to adsorb to the surface of the adsorbent. By analyzing the parameters obtained from the Freundlich isotherm, it is verified that the system has a heterogeneous surface, and the adsorption process is favorable since n has a value greater than one. It is also noted that other studies have presented the Langmuir isotherm as the best correlation in the adsorption of sodium diclofenac using activated carbon.^39,43^

The thermodynamic parameters (Table 5) indicate that the value of Δ*H*° is −39.77 kJ mol^–1^. This negative value indicates that the adsorption process is exothermic, which justifies a reduction in adsorption capacity with increasing temperature. The value of Δ*S*° is −0.17 kJ mol^–1^ K^–1^, in which this negative value indicates a reduction in randomness at the interface between adsorbed and dissolved diclofenac. The achieved negative values of Δ*G*° indicate that sodium diclofenac adsorption on activated carbon is spontaneous, confirming that the process has an exothermic nature, which is consistent with the data from the study on isotherms.

**Table 5:** Thermodynamic Parameters for Sodium Diclofenac Adsorption

*T* (K)	Δ*G*°_ads_ (kJ mol^–1^)	Δ*H*°_ads_ (kJ mol^–1^)	Δ*S*°_ads_ (kJ mol^–1^ K^–1^)
298	–8.38		
303	–7.73	–47.12	–0.13
308	–7.08		
313	–6.43		

These results reinforce that PET waste-derived activated carbon exhibits a high adsorption capacity and favorable adsorption thermodynamics, confirming its potential as a scalable adsorbent for environmental applications. Compared with conventional adsorbents, the material obtained in this study combines sustainability, low cost, and superior performance, highlighting its suitability for treating pharmaceutical effluents. Integrating physicochemical characterization with adsorption modeling provides a comprehensive understanding of the removal mechanism.

### 3.3 Process Simulation

The simulations performed in the Aspen Plus software were developed based on the combination of the kinetic model and the adsorption isotherm model resulting in the best fits (evaluated by the highest *R* ^2^ value), namely the pseudo-second-order and Langmuir models, respectively. The simulation of a transient condition is illustrated in Figure 6, which enables to verify that implementing a customized kinetic model in the software can accurately represent both the kinetic and equilibrium tendencies of the experimental data. Therefore, the application of the adsorption process, analogous to a chemical reaction, was validated, enabling the simulation of a continuous process.

**Figure 6:**
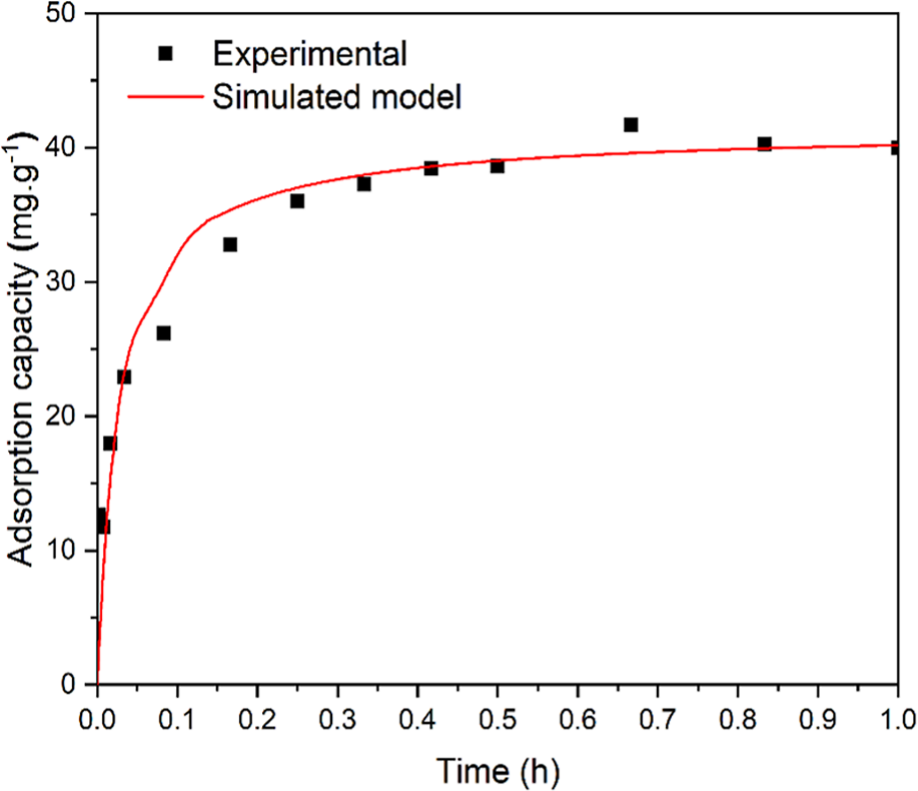
Adsorption capacity over time in the transient process: comparison between experimental data and simulated model.

The continuous adsorption column was simulated based on the analogy of a tubular reactor filled with adsorbent. The sensitivity analysis results were illustrated in Figure 7A,B (2D contour and 3D surface). The results were analyzed in terms of the percentage of adsorbed sodium diclofenac, taking into account the continuous nature of the process, based on the evaluation of mass flow rates. It is possible to check that low masses of adsorbent lead to higher percentages of adsorption capacities, which might seem counterintuitive. Until around 500 kg of adsorbent, these values are very similar, with a percentage higher than 0.2% ·kg^–1^, indicating that the amount of adsorbent can adsorb a proportional percentage of sodium diclofenac.

**Figure 7:**
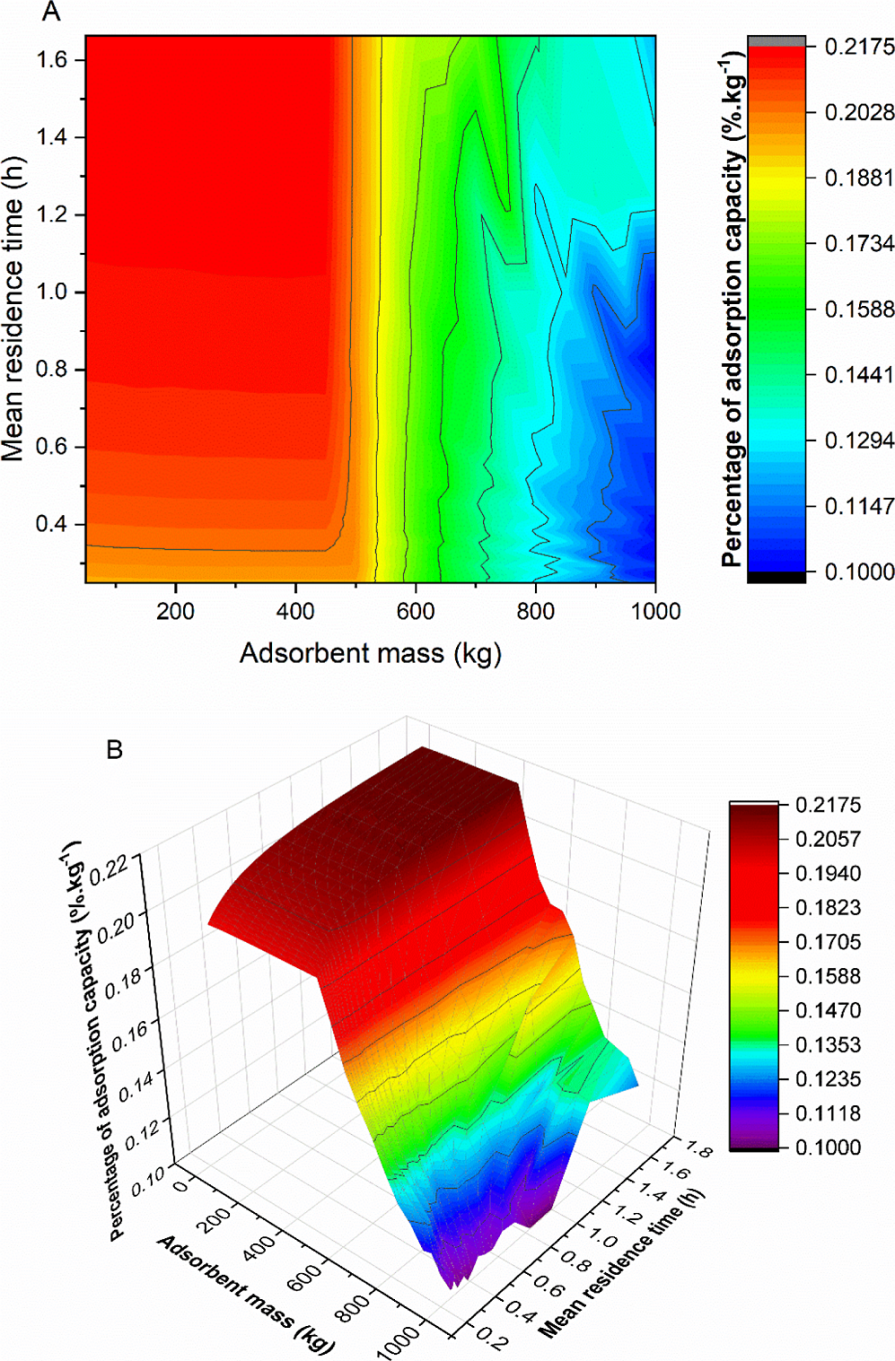
Percentage of adsorption capacity as a function of adsorbent mass and mean residence time in continuous process: (A) 2D contour and (B) 3D surface.

The percentage of diclofenac adsorbed per gram of adsorbent relative to the amount that could be adsorbed (if liquid phase removal were 100%) is high and relatively constant. This occurs because, with smaller adsorbent masses, each mass unit of adsorbent is responsible for removing a larger fraction of the available pollutant in the feed stream, thereby achieving a certain overall removal percentage. The adsorbent is, therefore, operating more efficiently in terms of its capacity utilized per mass, with the highest return (in terms of % liquid phase removal) for each gram of adsorbent used. Amounts of adsorbent exceeding 500 kg represent quantities that would not significantly increase the amount of sodium diclofenac adsorbed from the solution stream, resulting in a decrease in the adsorption capacity percentage.

Additionally, higher mean residence times tend to increase the percentage of adsorption capacity, which is expected since lower flow rates allow for a longer contact time between the solution containing sodium diclofenac and the adsorbent. However, considering the maximum of 500 kg of adsorbent, it is possible to observe that the percentage of adsorption capacity does not change significantly for mean residence times higher than 0.6 h, indicating that the adsorbent approaches saturation.

Based on the presented results, the adsorption column should operate with mean residence times of around 0.6 h and an adsorbent mass of up to 500 kg. However, it is essential to emphasize that the continuous study presented here is preliminary to prospecting some process conditions without oversizing the adsorption column. This fact opens the possibility of future investigations to evaluate other aspects, such as analyzing the breakthrough curve and regeneration of the adsorbent after saturation, which could be combined with an economic analysis of the process.

The simulation strategy adopted in this study goes beyond a conventional kinetic fit, since it enables the projection of process performance under continuous operation. The analogy between adsorption and catalytic reaction modeling in Aspen Plus allowed an estimation of a packed column’s adsorption capacity and operating conditions. Although preliminary, this approach provides valuable insights for scale-up, aligning the study with industrial practices of process design and optimization.

### 3.4 Comparison with Other Adsorbents

The activated carbon from PET waste was compared with the other adsorbents regarding treatment efficiency in removing sodium diclofenac (Table 6). The results demonstrate the material’s efficiency in removing this compound compared to other adsorbents. The adsorption capacity estimated in this study (200 mg·g^–1^) significantly exceeds that of several different materials, including biochars, composites, and modified polymers.

**Table 6:** Adsorbent Comparison

adsorbent	*q*_e_ (mg·g^–1^)	reference
HDBAC	125.55	^47^
N,S-containing hyper-cross-linked polymers	140.50	^48^
rice husk biochar	24.33	^49^
ZNA	85.90	^50^
LF-PPySAc	23.29	^51^
chitosan/fibrous silica KCC-1 composite	142.01	^52^
MSoGO@Bn	55.02	^53^
iron–manganese oxide composite	138.80	^54^
PET waste-derived activated carbon	200.00	this work

Among the high-performance materials, chitosan/fibrous silica KCC-1 composites (142.01 mg·g^–1^) and hyper-cross-linked N,S-containing polymers (140.5 mg·g^–1^) have demonstrated noteworthy adsorption capacities, primarily due to their high surface area and the presence of functional groups favorable for electrostatic interactions and hydrogen bonding with the diclofenac molecule. Similarly, inorganic composites, such as iron–manganese oxides (138.8 mg·g^–1^), benefit from redox-active surfaces that enhance their adsorptive affinity. These materials often require multistep synthesis, specialized reagents, or expensive precursors. In contrast, the activated carbon derived from PET waste presented in this study exhibited superior adsorption performance. It employed a low-cost and environmentally friendly precursor, reinforcing its potential for scalable and sustainable wastewater treatment.

The high adsorption capacity observed can be attributed to the morphological and chemical properties conferred by phosphoric acid activation, including the formation of mesopores and the enrichment of the surface with oxygen-containing functional groups, as well as a phenolic group, which facilitates π–π interactions and hydrogen bonding with the aromatic and carboxylic groups of diclofenac. Furthermore, the material’s low crystallinity and high porosity contribute to enhanced accessibility and diffusion of diclofenac molecules into the adsorbent matrix.

## 4 Conclusion

Through this study, it was possible to verify the efficiency of obtaining activated carbon from PET packaging and using the adsorbent to treat effluents containing sodium diclofenac. Studies have shown that the process occurs in monolayers and is a chemisorption process, making it irreversible. It was also observed that the process is exothermic, and efficiency decreases with increasing temperature.

The findings demonstrate that activated carbon derived from PET waste is an efficient and sustainable adsorbent for diclofenac removal, with a maximum adsorption capacity estimated as 200 mg·g^–1^. The adsorption process was shown to be exothermic, spontaneous, and better described by the Langmuir isotherm and pseudo-second-order kinetics. Integrating experimental data with Aspen Plus simulations enabled the projection of batch and continuous adsorption processes, providing an industrially relevant framework that is rarely explored in the literature. This work advances the state of the art by combining circular economy concepts, high-performance adsorption, and process modeling.
